# Relief from incidental fear evokes exuberant risk taking

**DOI:** 10.1371/journal.pone.0211018

**Published:** 2019-01-24

**Authors:** Sonja van Well, John P. O’Doherty, Frans van Winden

**Affiliations:** 1 CREED – Amsterdam School of Economics, and Amsterdam Brain and Cognition, University of Amsterdam, Amsterdam, The Netherlands; 2 Division of the Humanities and Social Sciences, and Computation and Neural Systems Program, California Institute of Technology, Pasadena, California, United States of America; Texas A&M University, UNITED STATES

## Abstract

Incidental emotions are defined as feelings that are unrelated to a decision task at hand and thereby not normatively relevant for making choices. The precise influence and formal theoretical implications of incidental emotions regarding financial risk taking are still largely unclear. An effect of incidental emotion on decision-making would challenge the main extant formal theoretical economic models because such models do not allow for an effect of incidental emotions. As financial risk taking is pervasive in modern economies, the role of incidental emotions is an important issue. The goal of this experimental study is threefold. First, we examine the impact of incidental fear on the choice between a sure and a risky monetary option. A well-validated method of fear induction, using electric shocks, is employed for that purpose. Based on emotion studies we hypothesize less risk taking under fear and more risk taking when relieved of fear. Our second goal is to investigate the relative performance of the main existing formal theoretical economic models (based on Expected Utility Theory, Prospect Theory, or the Mean-Variance model) in explaining the behavioral data. We also investigate how these models can be adjusted to accommodate any observed influence of incidental emotion. For that reason, we first theoretically model the potential pathways of incidental fear (and the relief thereof) via the valuation of the choice option rewards or risk-assessment. We then estimate the relevant parameters allowing for both additive as well as interactive effects. Our third and final goal is to explore the neural basis of any observed influence of incidental emotions on decision-making by means of a model-based fMRI analysis, using the findings of existing neuroeconomic studies as the basis for our hypotheses. Our results indicate that the relief of fear can give a substantial boost to financial risk taking (suggestive of exuberance). This impact is best captured by Prospect Theory if we allow for an increase in participants’ valuation of option outcomes when relieved of fear. Moreover, this impact is manifested at the neural level by the activity of the ventromedial prefrontal cortex (vmPFC), a brain area widely regarded as being central for valuation.

## Introduction

“Economists cannot avoid being students of human nature, particularly of exuberance and fear”, according to Alan Greenspan, former Chairman of the Federal Reserve Board of the US (see [1] p. 17). In his view, the emotions of fear and exuberance (a feeling of joyfulness) play a significant role in the development of stock market prices, with the former depressing and the latter boosting prices. The acknowledgment that emotions are important in financial decision-making, such as dealing with investment in risky assets, is steadily growing [2–6]. This is supported by mounting experimental evidence (for reviews [7–9]). In economics, typically, the emotions that have been studied are ones that are integral to the financial decision process, that is, feelings that arise and should objectively [8] or normatively [9] count as input when deciding between investments. Examples are anxiety about the resolution of an investment risk and the regret or disappointment that one will experience in case of a bad outcome. These integral emotions have been studied both from an experimental and formal theoretical modeling point of view (see [7–9]). Less attention has been paid to the impact of incidental emotions, that is, feelings at the time of decision that are not objectively or normatively relevant for deciding [8, 9]. Although some econometric studies have reported a correlation between stock returns and naturalistic phenomena such as the amount of sunshine or yearly seasons [10], controlled experimental investigation and formal modeling of the impact of incidental emotions on risk taking has been neglected [11].

This study focuses on a case of incidental fear and the relief thereof. It has been proposed that emotional states such as fear and the pleasant feeling of relief from fear could manifest themselves in the valuation and risk assessments of choice options [7–9]. In line with Greenspan’s view, this may respectively lead to less or more risk taking. Lerner and Keltner [12] found that individuals exhibiting dispositional fear showed pessimistic risk estimates and risk-averse choices. In contrast, individuals categorized as being dispositionally angry (as well as happy) were found to exhibit optimistic risk estimates and risk-seeking choices. At this stage it is largely unclear, however, what role experimentally induced incidental fear (and the relief thereof) might play in financial decision-making, and whether the main extant formal economic models—based on Expected Utility Theory (EUT), Prospect Theory (PT) or the Mean-Variance model (MV)–can accommodate any such effects. If it is found that incidental emotions do impact on financial decisions, then such findings would challenge these existing formal economic models because of the rational consequentialist perspective of the theories they are based on. In view of the pervasiveness of financial risk taking in economies, the role of incidental emotions is thus an important issue.

To enable a controlled investigation, we experimentally induced fear using a classical conditioning paradigm in which arbitrary cue stimuli were paired repeatedly with either an electric shock or the absence of shock. These cues were presented while participants had to choose repeatedly between a safe monetary option and a lottery, after learning that the possibility of a shock was unrelated to the choice they made. The goal of this study was threefold. First, we wanted to examine the influence of incidental fear on financial risk taking, using a well-validated method of fear induction [13,14]. We hypothesized that we would find evidence for less risk taking under fear and more risk taking when relieved of fear. Second, we aimed to investigate the relative performance of the main existing formal models (EUT, PT, MV) in capturing the behavioral data and aimed to determine whether these models can be adjusted to accommodate any observed impact of incidental fear. To achieve this, we theoretically modeled the potential ways in which incidental fear could impact the choice process. This could occur via an effect of incidental fear on the valuation of the choice options or alternatively via an effect of incidental fear on risk-assessment. Either of those effects could also potentially manifest as additive or as interactive effects [15]. Finally, this study was designed to explore the neural basis of the influence of incidental fear on valuation and risk-assessment, using a model-based fMRI analysis. Based on substantial prior evidence [16–20], we expected that the insula would be involved in risk-assessment, and that the ventromedial prefrontal cortex (vmPFC) would be involved in valuation. Depending on whether incidental fear or relief from fear was found to influence valuation processes or risk processes at the behavioral level, we hypothesized that a similar effect would manifest in the brain. That is, if fear is influencing valuation at the behavioral level, we expected that this would influence activation in valuation areas such as vmPFC, whereas if fear and/or relief is influencing risk-assessment at the behavioral level, we hypothesized that fear or its relief would influence activation in risk-responsive brain areas such as the insular cortex.

## Methods

### Participants

Thirty healthy undergraduate students (40% male) between the ages of 19 and 39 years (*M* = 23.3, *SD* = 4.2) participated in the study. All participants were right-handed, reported no psychiatric or neurological disorders and reported no current use of any psychoactive medication. Twenty-seven percent of the participants had participated in fMRI research previously, but none of the participants had previously taken part in a fear conditioning study.

The ethical committee of the University of Amsterdam approved the study protocol. All participants read and signed informed consent. Participants were paid a €25 show-up fee plus an additional amount (ranging from €0 to €80, *M* = 13.6, *SD* = 16.2) according to their decision (and the outcome) on one randomly selected trial.

### Measures and materials

#### Fear induction

We used a differential fear-conditioning paradigm with which participants were fear conditioned to a visual conditioned stimulus (CS) through multiple pairings with an aversive unconditioned stimulus (US). A control stimulus was paired with an affectively neutral US.

Unconditioned Stimuli (USs). We used a high and low electrical stimulation as the aversive versus neutral US (i.e., Shock and Touch, respectively). The intensity of the USs was adjusted individually. Accordingly, the Shock was set to a level defined by the participant as “uncomfortable, but not yet painful” (intensity range 4–18 mA, *M* = 7.7, *SD* = 3.1), whereas the Touch was set to a level defined as “tangible, but very comfortable” (intensity range 0.5–2 mA, *M* = 1.5, *SD* = 0.4). Level of intensity was significantly higher for the Shock as compared to the Touch (*t*(29) = 11.8, *p* <.001, *d* = 4.12).

Both USs were applied to participant’s right wrist and were delivered as a train of 5 pulses, with an inter-pulse interval of 20-ms. Spike duration was 5 ms and 2 ms for the Shock and Touch, respectively. Delivery of the USs was controlled by a Digitimer DS5 constant current stimulator (Welwyn Garden City, UK), through a pair of disposable MRI compatible carbon electrodes (Kendall H135TSG). Electrodes were pinched to a carbon electrode lead that was grounded through a radio frequency filter.

**Fig 1:**
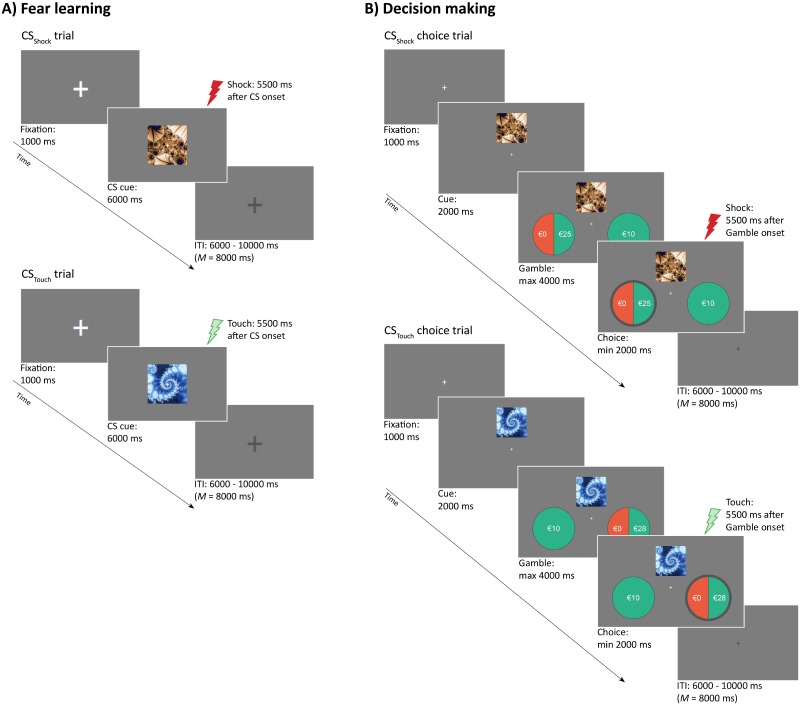
Experimental design. (**A**) Fear conditioning procedure. Participants were fear-conditioned to a visual conditioned stimulus (CS_Shock_) through multiple parings with an aversive unconditioned stimulus (Shock). A control stimulus (CS_Touch_) was paired with a neutral event (Touch). Reinforcement rate is 50%. (**B**) Decision-making task. Participants made a series of choices between a safe and risky option in the context of either a CS_Shock_ or CS_Touch_. Reinforcement rate is 30%. *Note*. Red and green lightning depicts the presentation of the Shock and Touch, respectively.

Conditioned Stimuli (CSs). Two images (a brown and blue fractal pattern), served as CSs (see Fig 1). Images were placed on a gray background and adjusted to a mean brightness. One of the images (i.e., CS_Shock_) was paired with the Shock, whereas the other image (i.e., CS_Touch_) was paired with the Touch (using 50% reinforcement rates). Assignment of the images as CS_Shock_ and CS_Touch_ was counterbalanced.

#### Decision-making task

The decision-making task consisted of a series of binary choices between a safe and a risky option. The safe option represented a guaranteed gain of €10. The risky option involved a lottery between a chance of a higher gain (ranging from €11 to €93) and a chance of no gain at all (€0). Note that all risky gambles are non-negative. See S3 Table for the complete set of risky options.

Two circles presented the choice options. Probability and magnitude of each option was represented by the size and color of the pie (see Fig 1). Green denoted a gain whereas red denoted no gain (i.e., €0). Presentation of the safe and risky options on the left or right side of the screen was counterbalanced.

#### Behavioral measures

Pleasantness Ratings. We used pleasantness ratings as a subjective measure of fear learning. Participants rated the pleasantness of each of the two CSs by using an 11-point Likert scale that ranged from 5 (*very pleasant*) to -5 (*very unpleasant*).

Pupil Size. In addition, we used the pupil dilation response to measure psychophysiological arousal (see e.g., [21]). Additionally, pupil dilation can also be seen as a psychophysiological index of human Pavlovian fear conditioning (see e.g., [22,23]). Pupil size was recorded continuously using a non-ferromagnetic eye tracker with fiber optic camera upgrade (EyeLink 2000, SR Research Ltd, Mississauga, Ontario, Canada). MRI compatible batteries powered the eye tracker. Data were sampled at 1000 Hz and transmitted to the host PC placed outside the scanner room. Raw EyeLink data were offline converted to ASCII using the EDF2ASC converter. Subsequently, samples around missing values (i.e., 50 ms before and after each series of 5 missing samples) were regarded as unreliable and were replaced by linear trend at point (see e.g., [24]). Data were then low-pass filtered (third-order Butterworth, 4Hz). Subsequently, pupil responding was calculated as the peak change from baseline (i.e. last 250 ms of ITI) in a window of 5.5 s and 2.0 s after CS cue onset for the fear-conditioning and decision-making phase, respectively. Trials that suffered substantial signal loss (i.e., 50% of relevant baseline or CS samples) were replaced completely by linear trend at point for CS_Shock_ and CS_Touch_ conditions separately. Finally, pupil dilation responses were normalized within participants for the fear-conditioning and decision-making phase, separately.

#### Neuroimaging

Imaging was conducted using a 3T MRI scanner (Philips, Achieva XT) with a 32-channel head coil. Whole-brain blood-oxygenation-level-dependent MRI images were acquired (GE-EPI; TR = 2000 ms; TE = 27.6 ms; FA = 76.1°; FOV = 240 mm; matrix 80 x 80; slice thickness = 3 mm; 37 axial slices sequentially acquired). Additionally, to allow for anatomical localization of functional activation, a T1-weighted anatomical image was obtained for each participant (TR = 8.2 ms; TE = 3.7 ms; FA = 8°; FOV = 240 mm; matrix = 240 x 240; slice thickness = 1 mm; 220 axial slices sequentially acquired).

Imaging data were processed and analyzed using FEAT (FMRI Expert Analysis Tool) version 6.00, part of FSL (FMRIB’s Software Library, www.fmrib.ox.ac.uk/fsl) and MATLAB (The MathWorks, Natick, MA). First, functional images were motion corrected (MCFLIRT [25]), slice time corrected (Fourier-space time series phase-shifting), spatially smoothed (5-mm full-width-at-half maximum Gaussian kernel), and high-pass filtered (cutoff = 100 s). Then, structural images were brain extracted (BET [26]). Subsequently, for each participant, functional images were aligned to the structural image using boundary-based registration (BBR [27]).

#### Post-experimental questionnaire

Retrospectively, participants rated the amount of fear and irritation that each of the CSs elicited before the experiment and during the two separate experimental tasks on an 11-point Likert scale that ranged from -5 (*not at all*) to 5 (*very much*).

### Procedure

Participants were informed about the upcoming scan session. They were told that the scan session would consist of two experimental tasks. Concerning the first task (fear-conditioning) participants were instructed to look carefully at the images that would be presented on the screen as one of the images would sometimes be followed by a Shock, whereas the other would sometimes be followed by a Touch. Participants were further instructed to learn the correct CS-US associations. Concerning the second task (decision-making) participants were instructed to make a series of decisions between a safe and risky option presented on the screen. They were instructed to decide as fast as possible within a 4 s response window. It was stressed that one of trials would be selected at the end of the experiment and that they, depending on their decision, would receive the actual outcome of that trial in cash.

Next, participants entered the scanner room, shock electrodes were attached and the intensity of the Touch and Shock was set. Subsequently, participants were placed in the scanner. Foam pads were placed around participants’ heads to minimize head movement during scanning. For attenuation of the scanner noise, participants wore earplugs and headphones. Throughout the scan session, stimuli and additional instructions were back-projected onto a translucent screen placed at the head of the scanner table. Participants viewed the screen by means of a mirror attached to the head coil.

Inside the scanner participants first performed a five-point calibration of the EyeLink eye tracker. Participants then rated the pleasantness of the CSs for the first time. After that, participants were exposed to the differential fear-conditioning procedure. During this fear induction phase, both the CS_Shock_ and CS_Touch_ were presented 15 times. See Fig 1A for a graphical representation of a typical fear learning trial. Each trial began with the presentation of an active fixation cross (1 s). Thereafter, each CS was presented for 6 s. Fifty percent of the CS presentations were reinforced by the US. US onset was delayed 5.5 s after CS onset. After CS offset, a passive fixation cross remained on the screen for the entire duration of the inter trial interval (ITI). The ITI’s varied between 6, 7, 8, 9, and 10 s (*M* = 8 s). Trial order and duration of the ITI were quasi-random, such that no more than three consecutive trials or ITIs were of the same type. To conclude the fear-conditioning phase, participants retrospectively rated the current pleasantness of the CSs.

Subsequently participants performed the decision making task, that was divided into 4 equal runs separated by a short self-paced resting period. In total, participants made a series of 168 binary choices between a safe and risky option in the co-occurrence of either a CS_Shock_ or a CS_Touch_. See Fig 1B for a graphical representation of a typical decision-making trial. Each trial started with the presentation of an active fixation cross (1 s) immediately followed by the onset of a CS cue (8 s). Gamble onset was delayed 2 s after CS cue onset. Choices were made within a 4 s window after gamble onset. Choices were immediately confirmed by means of visual feedback on the screen. The computer program recorded choices and reaction times. Half of the gambles was presented with the CS cue that signaled the Shock (i.e., CS_Shock_-Gamble) whereas the other half was presented with the CS cue that signaled the Touch (i.e., CS_Touch_-Gambles). To avoid extinction of the learned CS-US association, thirty percent of the CS-Gamble trials was reinforced by the US (US onset was delayed 7.5 s after CS onset). The use of a passive fixation cross, trial order and duration of ITI’s were similar to the fear-conditioning phase. Immediately after the decision-making task, participants rated the pleasantness of the CSs. Thereafter one decision-making trial was randomly selected and the outcome was presented on the screen for payout.

Furthermore, outside the scanner room, participants completed the post-experimental questionnaire and were paid and debriefed. The laboratory visit took about 2.5 hr.

### Analyses

#### Fear induction

Behavioral measures. Behavioral measures were analyzed using the SPSS software (version 21.0.0.2, IBM Corp, USA). For all behavioral measures, where appropriate, Greenhouse-Geisser corrections were applied to control for the violation of the sphericity assumption.

Pleasantness Ratings. To confirm successful fear learning on the subjective level, we analyzed the pleasantness ratings using a repeated measures ANOVA with CS (CS_Shock_ vs. CS_Touch_) and phase (baseline, after fear-conditioning, vs. after decision-making) as within-subjects variables.

Pupil Size. We used a similar set of CS x Phase repeated measures ANOVAs to examine successful fear learning on the psychophysiological level (i.e., pupil dilation). Here, the within-subjects variable phase comprised early (trials 1–5), mid (trials 6–10), versus late (trials 11–15) acquisition or early (trials 1–84) versus late (trials 85–168) decision-making.

#### Decision-making: Behavior, models, and model-based fMRI analysis

Choice behavior. For the analysis of choice behavior we used the SPSS software (version 21.0.0.2, IBM Corp, USA). First, we compared choice frequency (percentage of trials where the Safe option was chosen) between the CS_Shock_- and CS_Touch_-trials, using paired *t*-tests. Similarly, we examined differences on the CS_Shock_-versus CS_Touch_-trials in the expected value of the choices (i.e., chosen Expected Value (cEV)) and reaction times.

Modeling choice behavior and parameter estimation. To enable a model-based fMRI analysis, we estimated and compared the fit of different economic models, using STATA (version 13.1, StataCorp, USA). We took the Prospect Theory (PT) model [28,29] as a starting point in our formal modeling approach because of its behavioral experimental support (see e.g., [30]), its distinction between valuation and probability weighting, and the fact that it embeds the standard Expected Utility model as a special case.

In the current study participants had to choose between two prospects (see S3 Table): a Safe prospect (S) guaranteeing an outcome (x_S_) of 10 with a probability (p_S_) of 1 (S: x_S_ = 10, p_S_ = 1), and a Risky prospect (R) giving a non-negative outcome (x_R_) with a positive probability (0 < p_R_ < 1) (R: x_R_, p_R_; 0, 1—p_R_). For the valuation of x, we use a standard power function, with *valuation parameter* α:

$$\text{v}\left(\text{x}\right)={\text{x}}^{\alpha }.$$ (1)

Expected PT-utility (U_PT_) is defined by:

$${\text{U}}_{\text{PT}}\left(\text{x}\right)=\text{w}\left(\text{p}\right)\cdot \text{v}\left(\text{x}\right),$$ (2)

where the probability weighting function w(p) is specified as [31,32]:

$$\text{w}\left(\text{p}\right)=\delta {\text{p}}^{\gamma }/\left[\delta {\text{p}}^{\gamma }+\;{\left(1-\text{p}\right)}^{\gamma }\right].$$ (3)

The advantage of this specification is that it accounts well for individual heterogeneity [33] and has easily interpretable parameters that are of particular interest here. The parameter γ influences the slope of the weighting function and measures sensitivity towards changes in probability. If linear weighting (with γ = 1) stands for rationality, then γ can be seen as an *index of rationality* [34]. The parameter δ influences the elevation of the weighting function and can be seen as an *index of pessimism* because a lower δ implies a lower weight for every probability. Assuming a (soft-max) logit choice model, and letting

$$\Delta {\text{U}}_{\text{PT}}={\;\text{U}}_{\text{PT}}\left({\text{x}}_{\text{S}}\right)-{\text{U}}_{\text{PT}}\left({\text{x}}_{\text{R}}\right),$$ (4)

the probability of choosing the Safe option is given by:

$$\text{Pr}\left(\text{S}│{\text{U}}_{\text{PT}}\left({\text{x}}_{\text{S}}\right),{\text{U}}_{\text{PT}}\left({\text{x}}_{\text{R}}\right)\right)=\text{exp}\left(\Delta {\text{U}}_{\text{PT}}/\theta \right)/\left[1+\text{exp}\left(\Delta {\text{U}}_{\text{PT}}/\theta \right)\right],$$ (5)

where θ is the noise (inverse choice intensity) parameter.

This PT model offers a natural way to allow for the potential influences of the incidental emotion of fear that were discussed in the Introduction, as α can capture a valuation effect (i.e., an effect on v(x), see (Eq 1)), whereas the parameters γ and δ can capture a probability weighting effects (related to rationality and pessimism, respectively). Note, however, that the hedonic value of the emotional state triggered by the CS, say f, need not necessarily affect utility in an interactive way, which formally implies:

$${\text{U}}_{\text{PT}}\left(\text{x}\right)=\text{w}\left(\gamma \left(\text{f}\right),\delta \left(\text{f}\right)\right)\cdot {\text{x}}^{\alpha (\text{f})}.$$ (6)

Alternatively, f may influence utility in the following additive way [15]:

$${\text{U}}_{\text{PT}}\left(\text{x}\right)=\text{w}\left(\text{p}\right)\text{v}\left(\text{x}\right)+\text{f},$$ (7)

in which case it is easily seen that its effect on choice behavior would cancel out, as the utility obtained from both the Safe and the Risky option is equally affected.

Note also that the expected utility (EU) model is embedded in this PT model, requiring that γ = δ = 1 (and, thus, w(p) = p) and arguably α = 1 [35]. Consequently, this model cannot formally accommodate a valuation effect nor a probability weighting effect from the incidental emotion of fear.

Finally, we consider the Mean-Variance (MV) model, a key model from finance which is related to the EU model (see e.g., [36]), specified as:

$${\text{U}}_{\text{MV}}\left(\text{x}\right)={\text{b}}_{1}\text{EV}\left(\text{x}\right)-{\text{b}}_{2}\text{VAR}\left(\text{x}\right),$$ (8)

where the first term. with EV(x) = px, represents the Mean component and the second term, with VAR(x) = p(1-p)x^2^, represents the Variance component. The probability of choosing the Safe option is further formalized as above, with U_PT_ substituted by U_MV_. Strictly, b_1_ = 1 in this model, but if we allow b_1_ ≠ 1 this model can formally capture a valuation effect in addition to a risk assessment effect via b_2_. Note that an additive effect of f on utility, instead of an interactive effect, would leave choice behavior unaffected, as was the case for the PT model.

To compare the model-fit of different models we used the Akaike’s Information Criterion (AIC) and Bayesian Information Criterion (BIC). AIC and BIC are likelihood information criteria that penalize for the number of parameters in a model and are widely used for model selection [37]. Both information criteria give a measure of uncertainty for each model where lower values indicate a better model-fit.

Linking brain activation to model parameters. To further examine the influence of incidental emotions on choice behavior, we explored differential brain activation during CS cue presentation (contrast CS_Shock_ versus CS_Touch_) and tried to link this CS cue induced differential brain activation to differences in estimated model parameters. Due to problems with data storage, data of two participants had to be removed from the fMRI analyses.

For the imaging data, statistical analyses were carried out using a general linear model (GLM [38]). First, we performed a lower-level FEAT analysis on each of the four runs for each subject. Next, to combine the lower-level FEAT results from the four separate runs we performed a fixed-effects analysis for each subject. Finally, we performed across-subjects analyses using FSL’s FLAME (fMRIB’s Local Analysis of Mixed effects).

Because we were interested in differential CS cue activation, we modeled CS cue presentation with two regressors of interest (i.e., CueCS_Shock_ and CueCS_Touch_). Cue presentation was defined as the 2s period from CS cue onset (see Fig 1B) until the presentation of the gamble. We further added four regressors of no interest that modeled the decision period (from the onset of the choice trial until subject made their choice), CS presentation and delivery of the Shock and Touch. All regressors were convolved using a canonical hemodynamic response function. To identify regions of interest (ROIs) involved in differential CS cue processing we used contrast analyses (e.g., CS_Shock_ > CS_Touch_; CS_Touch_ > CS_Shock_), using a cluster-corrected height threshold of *p* <.005. Individual parameter estimates were then extracted for each ROI and each contrast, for linking differential brain activation to differences in estimated model parameters.

All tests are two-tailed, unless otherwise indicated. As statistical threshold *p* <.05 will be applied; effects with.05 < *p* <.10 will be referred to as marginally significant or trending toward significance.

## Results

Data from two participants were excluded from all analyses due to a lack of risky choices on the decision-making task (≤ 1% of the choices; one participant) or too many missed choices because of self-reported sleepiness (≥ 10% of the trials; one participant).

### Successful fear induction

**Fig 2:**
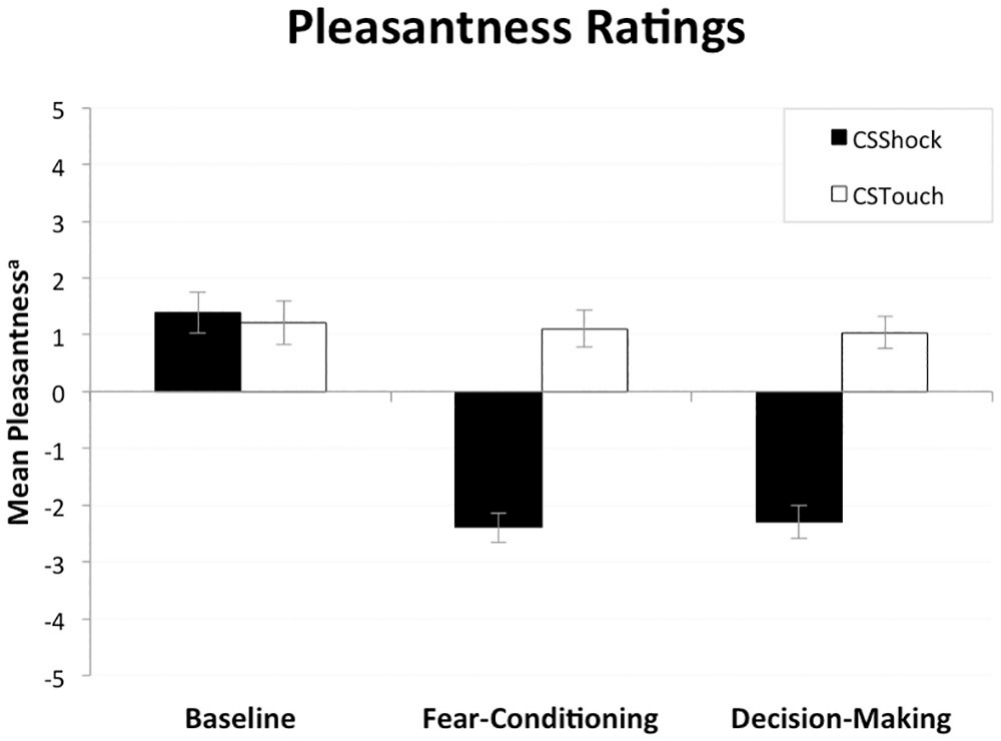
Mean pleasantness ratings for the fear-conditioned (CS_Shock_) and neutral (CS_Touch_) stimulus as a function of experimental phase (i.e., baseline, after fear conditioning, and after the decision-making task). Error bars depict standard error of the mean. ^a^5 = *very pleasant*; -5 = *very unpleasant*.

Successful fear induction was evident from the subjective pleasantness ratings for the fear-conditioned (CS_Shock_) and neutral (CS_Touch_) stimulus during the different experimental phases (baseline, after fear conditioning, and after the decision-making task; see Fig 2). Analyses of variance showed differential liking of the CSs over the course of the experiment (CS × Phase: *F*(2, 54) = 28.23, *p* <.001, *η*_p_^2^ = .51). As expected, pleasantness ratings of the CSs did not differ before the fear-conditioning phase (*t*(27) = .42, *p* = .679, n.s.), but after the fear-conditioning phase participants reported lower pleasantness for the CS_Shock_ as compared to the CS_Touch_ (*t*(27) = 7.45, *p* <.001, *d* = 1.41). This differential liking of CSs sustained after the decision-making phase (*t*(27) = 6.92, *p* <.001, *d* = 1.31).

**Fig 3:**
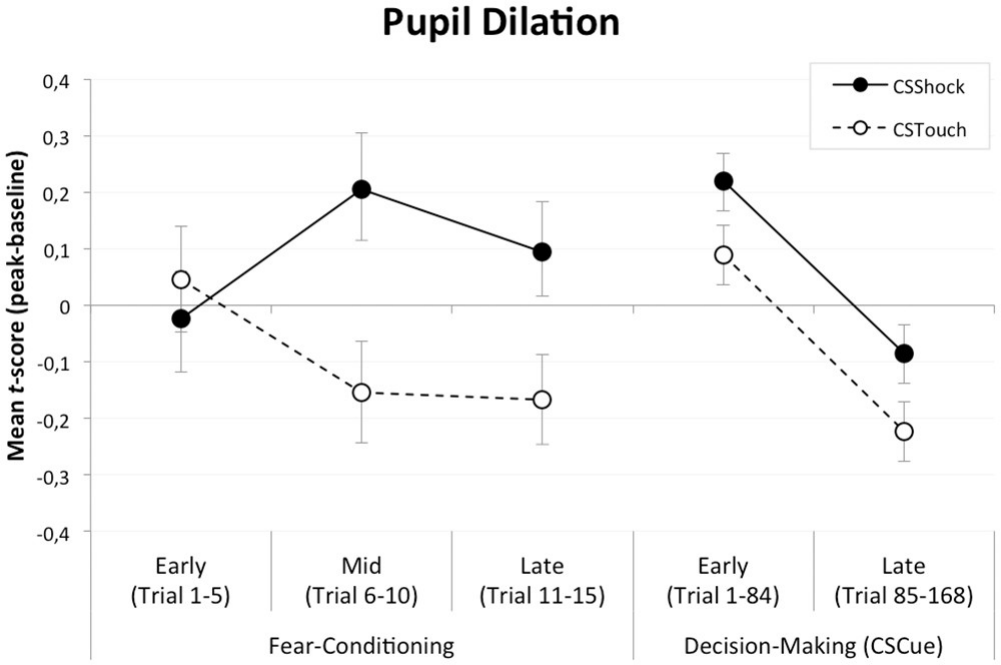
Mean pupil dilation response (*t*-score: Peak change from baseline) for the fear-conditioned (CS_Shock_) and neutral (CS_Touch_) stimulus across fear conditioning (i.e., early, mid, to late acquisition) and CS cue presentation during the decision-making task (i.e., early vs. late). Error bars depict standard error of the mean. Note. CS = Conditioned Stimulus.

Successful fear learning was also suggested by the psychophysiological expression of arousal as indexed by the pupil dilation response (Fig 3). For the fear-conditioning phase, analysis of variance showed a marginally significant increase in differential pupil responding (CS x Phase; *F*(2, 54) = 2.74, *p* = .073, *η*_p_^2^ = .09). As expected, the fear-conditioned stimulus (CS_Shock_) elicited larger pupil dilation responses than did the neutral stimulus (CS_Touch_) during mid acquisition (*t*(27) = 2.46, *p* = .021, *d* = 0.47) and late acquisition (*t*(27) = 2.20, *p* = .036, *d* = 0.47), but not during early acquisition (*t*(27) = 0.48, *p* = .636, n.s.). Indicating that over the course of the fear-conditioning phase, participants learned to fear the CS_Shock_ more than the CS_Touch_. Furthermore, as expected, analyses of variance on pupil responding during CS cue presentation throughout the decision-making phase [39] revealed a main-effect trending toward significance of CS type (CS; *F*(1, 26) = 3.28, *p* = .082, *η*_p_^2^ = .11) as well as a main-effect of phase (*F*(1, 26) = 22.35, *p* <.001, *η*_p_^2^ = .46). That is, although participants showed diminished pupil dilation to the presentation of the CS from the beginning to the end of the decision making phase, participants showed sustained differential pupil responding to the presentation of the CS (CS_Shock_ > CS_Touch_). This indicates that participants maintained a conditioned fear response to the CS_Shock_ more than the CS_Touch_ throughout the entire decision-making phase.

Thus, both the subjective pleasantness ratings and the pupil dilation responses suggest that, as intended, throughout the experiment participants perceived the affective quality of the CSs differently.

### Evidence for impact on choice behavior

Contrary to our initial expectations, we found no significant difference between the choices during CS_Shock_-trials and CS_Touch_-trials (*t*(27) = 0.06, *p* = .953, n.s.). To further explore a CS effect, we examined choices after a reminder of the learned CS-US relation. It could be reasoned that the CS-US association is temporarily strengthened and has more effect immediately after the re-exposure of a CS-US pairing. Then, observing a CS_Shock_ cue, when having just experienced a Shock (US_Shock_) after a CS_Shock_ cue, would lead to an excitation of learned fear (a strengthened CS_Shock_-US_Shock_ association), with less risk taking as hypothesized consequence. On the other hand, while observing a CS_Touch_ cue, when having just experienced a Touch (US_Touch_) after that type of cue, would lead to an excitation of learned safety (a strengthened CS_Touch_-US_Touch_ association) and a larger inhibition of fear [40]. As hypothesized, this would in turn induce more risk taking. Accordingly, we examined trials immediately after the delivery of a Shock, a Touch, or no electro stimulation, to check whether a differential behavioral response to the CS cues occurred.

**Fig 4:**
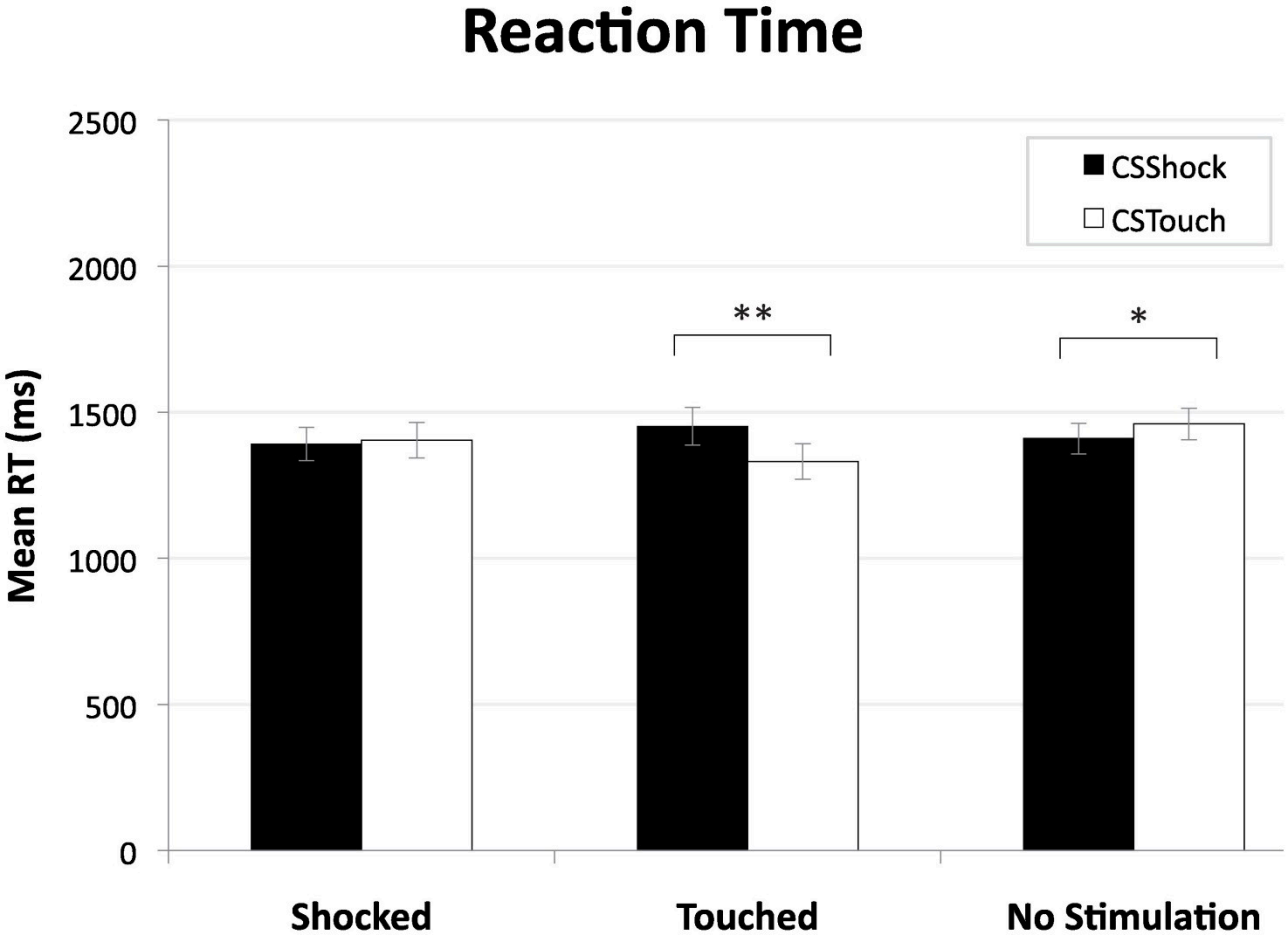
Mean reaction time (in ms) for CS_Shock_- and CS_Touch_-trials as a function of reminder (i.e., proceeding a trial in which a Shock, a Touch, or no electro stimulation was given). Error bars depict standard error of the mean. *Note*.*p <.05, **p <.005.

Our first piece of evidence of a differential CS effect concerns reaction times. Analyses of variance revealed a highly significant CS by preceding reminder type interaction (*F*(2, 54) = 7.63, *p* = .001, *η*_p_^2^ = .22); see Fig 4. That is, after the delivery of a Touch, participants were faster to make a decision on succeeding CS_Touch_-trials than on CS_Shock_-trials (*t*(27) = 3.23, *p* = .003, *d* = 0.61). Additionally, without electro stimulation on the previous trial, participants responded significantly slower to succeeding CS_Touch_-trials as compared to CS_Shock_-trials (*t*(27) = -2.60, *p* = .015, *d* = 0.49). The delivery of a Shock did not elicit differential reaction times for succeeding CS_Shock_- and CS_Touch_-trials (*t*(27) = 0.30, *p* = .771, n.s.).

**Fig 5:**
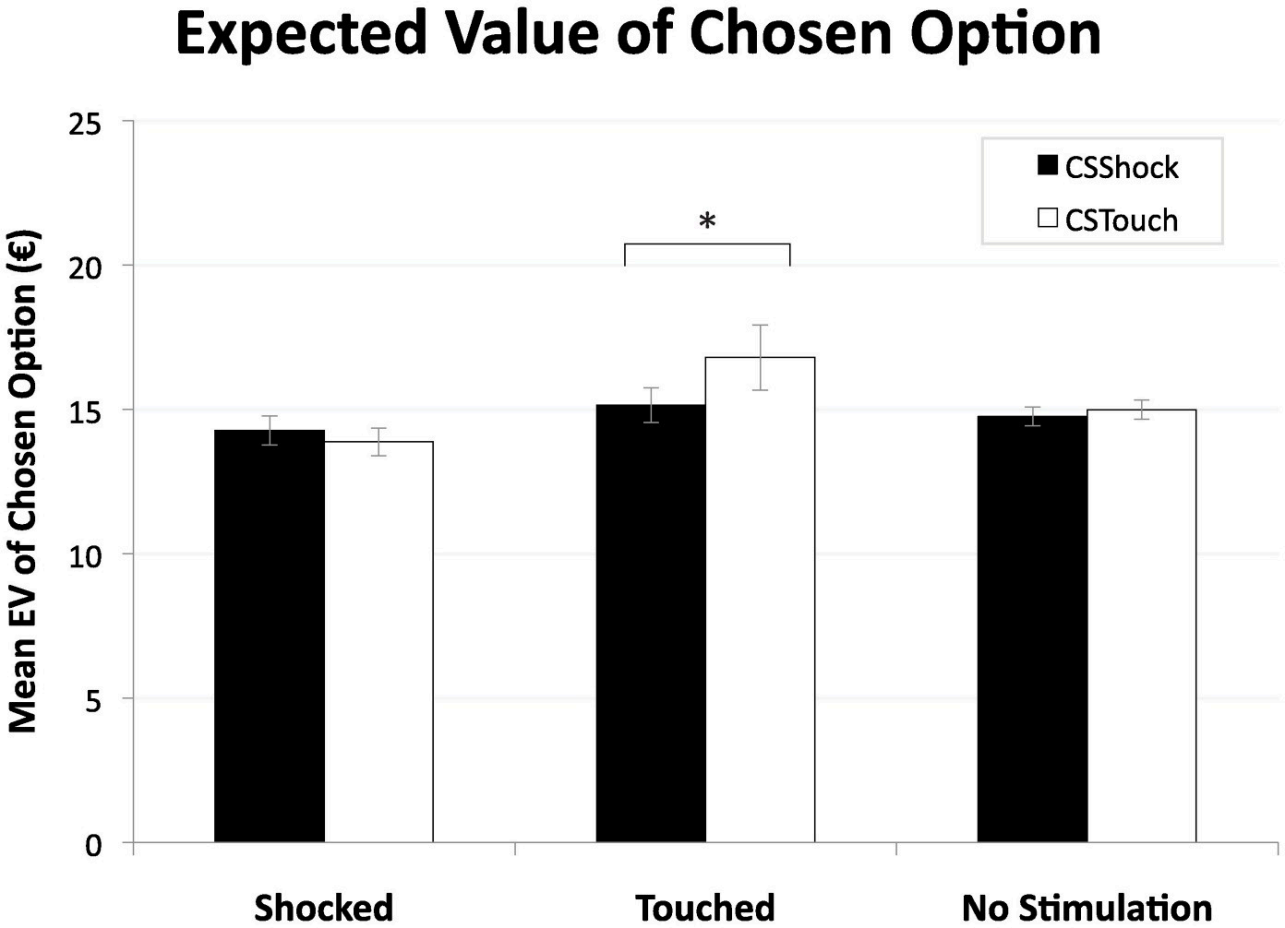
Mean expected value (in Euros) of the chosen option for CS_Shock_- and CS_Touch_-trials as a function of reminder (i.e., proceeding a trial in which a Shock, a Touch, or no electro stimulation was given). Error bars depict standard error of the mean. *Note*. *p <.05.

A second piece of evidence comes from the expected value of the chosen option (cEV). Overall, analyses of variance on cEV revealed an interaction effect of CS by preceding reminder type (*F*(1.35, 36.41) = 2.67, *p* = .100, *η*_p_^2^ = .09, ε = .674) that is only at the brink of being marginally significant. Participants’ choices were affected by the CS, only after the delivery of a Touch, in the hypothesized direction of more risk taking with a higher expected value on succeeding CS_Touch_-trials as compared to CS_Shock_-trials (*t*(27) = 1.92, *p* = .032 (one-tailed), *d* = 0.44); see Fig 5. This increase in expected value was also economically significant as it amounted to 11%. The delivery of a Shock or no stimulation, however, did not elicit a differential CS effect on the cEV of succeeding trials (*t*(27) = 0.76, *p* = .452, n.s., and *t*(27) = 1.25, *p* = .222, n.s., respectively).

Our findings indicate that, for behavior to be affected by incidental fear, it is not enough to have learned an association between some cue and (the absence of) a fearful future event. Only after a reminder in the form of a re-exposure to the event itself (the unconditioned stimulus) were behavioral effects detected. Specifically, we found that participants, after having been exposed to a neutral (Touch) reminder, exhibited faster choice reaction times and took more risk with a higher expected value when exposed to the CS_Touch_ cue as compared to the CS_Shock_ cue.

This *relief-of-fear effect* cannot be accounted for by standard formal economic models (EUT, PT, MV). We will now show that it can be accounted for by allowing the parameters of these models to be condition dependent. Estimation of these adapted models will show further evidence of a differential CS effect. Moreover, developing such adapted models enables us to discriminate whether this effect is due to changes in valuation and/or risk perception, as well as to assess the comparative performance of these models in explaining the data. These next steps will further prepare the way for a subsequent model-based fMRI analysis to test for evidence of a neural substrate for our finding.

### Prospect Theory adjusted for valuation effect best explains behavioral effects

**Fig 6:**
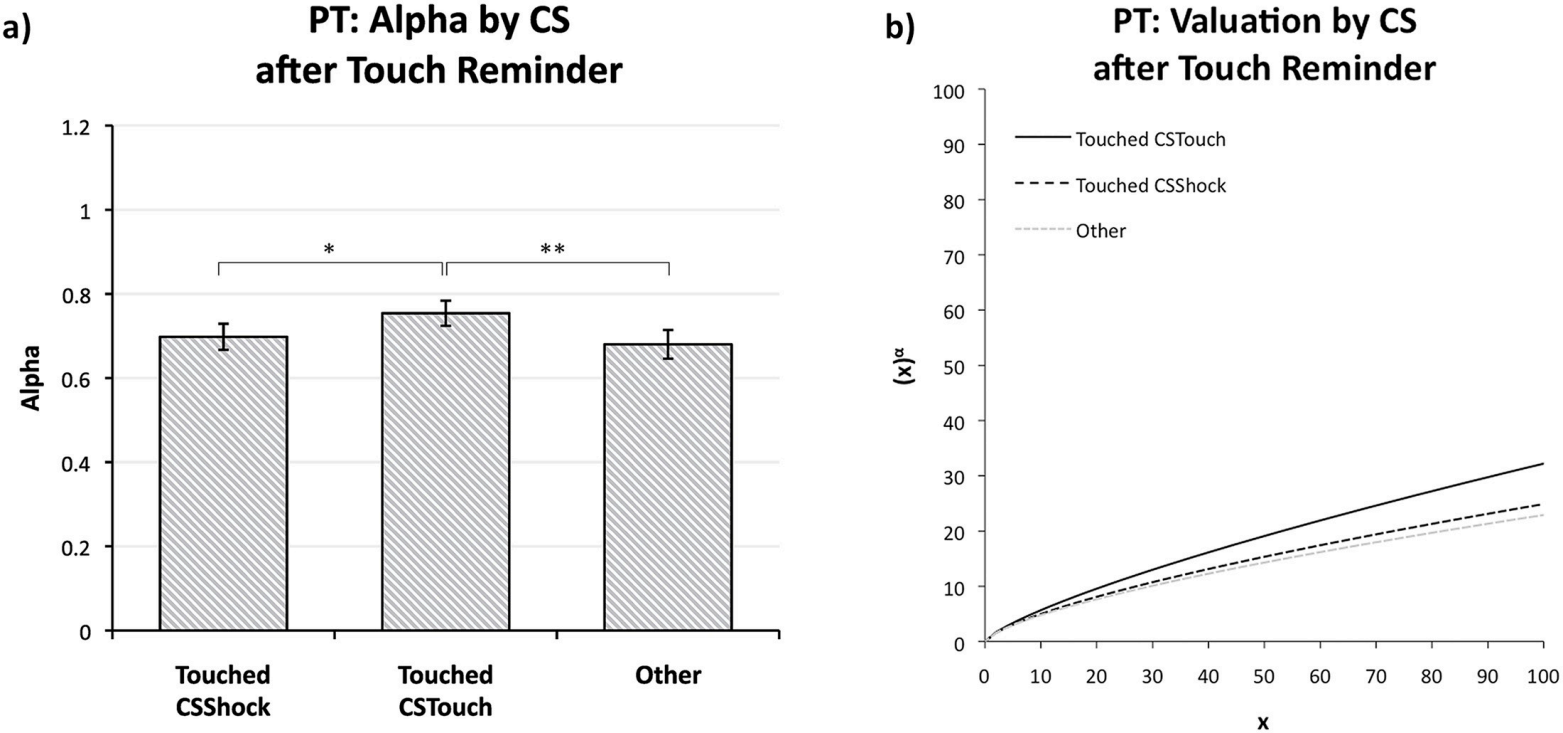
Estimates for the adapted PT-model. Plot (**A**) depicts estimates for the alpha (*α*) coefficient after a Touch reminder. Plot (**B**) depicts subjective valuation after a Touch reminder. *Note*. PT = Prospect Theory; CS = Conditioned Stimulus. *p <.05, **p <.005.

Regression analysis on choice behavior across all participants and trials, based on the different economic models tested (EUT, PT, MV), revealed a Reminder x CS type of interactive effect of incidental fear (relief) affecting the model parameters (see Methods and S1 and S2 Tables). Moreover, a model comparison showed that a version of the PT model with a condition-specific value function parameter (*α*) for CS_Shock_ versus CS_Touch_ trials after a Touch reminder performed best (using two standard criteria for model comparison: AIC and BIC; see Methods and S1 and S2 Tables). Specifically, Touched-CS_Touch_ trials revealed a higher α estimate than Touched-CS_Shock_ trials (0.755 and 0.700, respectively; *Χ*^2^(1, 28) = 3.63, *p* = .028 (one-tailed)); see Fig 6A. Furthermore, after a Touch reminder, CS_Touch_ but not CS_Shock_ trials showed an increase in the α estimate as compared to the remaining trials (0.755 vs. 0.679 (*χ*^2^(1, 28) = 8.41, *p* = .002 (one-tailed)) and 0.700 vs. 0.679 (*χ*^2^ = 0.83, *p* = .363, n.s., respectively). These results point to a relief-of-fear effect that manifests via an increased valuation of outcomes; see Fig 6B. In further support of a valuation effect, no effect was found for the parameters of the probability weighting function of the PT model, which besides the standard parameter (*γ*) influencing its curvature included a level-related parameter (*δ*) for pessimism or optimism (see Methods and S1 Table). Incidentally, the estimates of the unconditioned parameters (*α*, *γ*, *δ*) are all within the range of estimates that are found in the literature (for an overview, see [41, 42]).

After a Shock reminder we found some evidence for an improvement in model fits via an increase in γ for Shocked-CS_Shock_ trials (compared to the standard PT model), but only for the AIC measure while the γ estimate for Shocked-CS_Shock_ trials was only trending toward being significantly higher than for Shocked-CS_Touch_ trials (0.804 and 0.661, respectively; *Χ*^2^(1, 28) = 1.99, *p* = .079 (one-tailed)). Moreover, a combined model allowing for both a condition-dependent α and γ showed only some further improvement with respect to the AIC, but a deterioration regarding the BIC, while leaving the α-estimates unaffected (see S1 Table).

Consistent with our behavioral findings above, these estimation results also point at a *relief-of-fear effect*. Furthermore, this effect appears to be best captured by Prospect Theory once we allow for an *increase in valuation* in that condition. Applying the estimated PT model to our set of gambles predicts indeed more risk taking with higher expected value, as observed in Fig 5.

All in all, our evidence suggests a valuation effect of the relief of incidental fear, but no risk-assessment effect. Using individual-level estimates of the adapted PT model, we next investigate via a model-based fMRI analysis whether neural support for this valuation effect can be found.

### Evidence for an effect of incidental fear on valuation in the brain

Based on the modeling results, we used the adapted PT model, which allows for Reminder x CS dependency of its parameters. Therefore, we modeled CS cue presentation with six regressors of interest (i.e., Shocked_CS_Shock_, Shocked_CS_Touch_, Touched_CS_Shock_, Touched_CS_Touch_, NoStimulation_CS_Shock_, and NoStimulation_CS_Touch_). To localize brain areas exhibiting activation related to differential CS cue processing, we computed a Relief contrast, that is, Touched_CS_Touch_ > Shocked_CS_Shock_ and a Fear contrast, that is, Shocked_CS_Shock_ > Touched_CS_Touch_. For both contrasts we applied a cluster-corrected height threshold of *p* <.005. For both contrasts no cluster survived the whole-brain cluster-correction. At a more liberal height threshold of *p* <.005 (uncorrected), requiring a minimum cluster size of 20 voxels (extent-threshold), we observed differential CS cue processing (see Table 1). Regarding the Relief contrast, encountering the CS_Touch_ cue after a Touch reminder, relative to the CS_Shock_ cue after a Shock reminder, elicited more activation in the orbital frontal cortex (OFC), ventromedial prefrontal cortex (vmPFC), parahippocampal gyrus, and occipital fusiform gyrus. On the other hand, with regard to the Fear contrast, presentation of the CS_Shock_ cue after a Shock reminder, relative to the CS_Touch_ cue after a Touch reminder, only elicited greater activation in the parietal operculum cortex (see Table 1).

**Table 1:** Brain activation related to CS cue presentation (Touched_CS_Touch_ vs. Shocked_CS_Shock_).

Contrast Region of activation		Z max (mm)			*n* Voxels	*Z*
		*X*	*Y*	*z*		
Relief: Touched_CS_Touch_ > Shocked_CS_Shock_						
Orbital Frontal Cortex	R	20	36	-12	214	4.09
Ventro Medial PreFrontal Cortex		-10	50	-4	83	3.32
Parahippocampal Cortex	R	32	2	-38	32	3.43
Occipital Fusiform Gyrus	L	-26	-90	-10	24	2.97
Ventro Medial PreFrontal Cortex		-6	38	-6	24	3.26
Fear: Shocked_CS_Shock_ > Touched_CS_Touch_						
Parietal Operculum Cortex	L	-50	-34	24	32	3.60

*Note*. CS = Conditioned Stimulus; Coordinates are reported in MNI (Montreal Neurological Institute) space.

*p* <.005 (uncorrected), requiring a minimum of 20 voxels.

**Fig 7:**
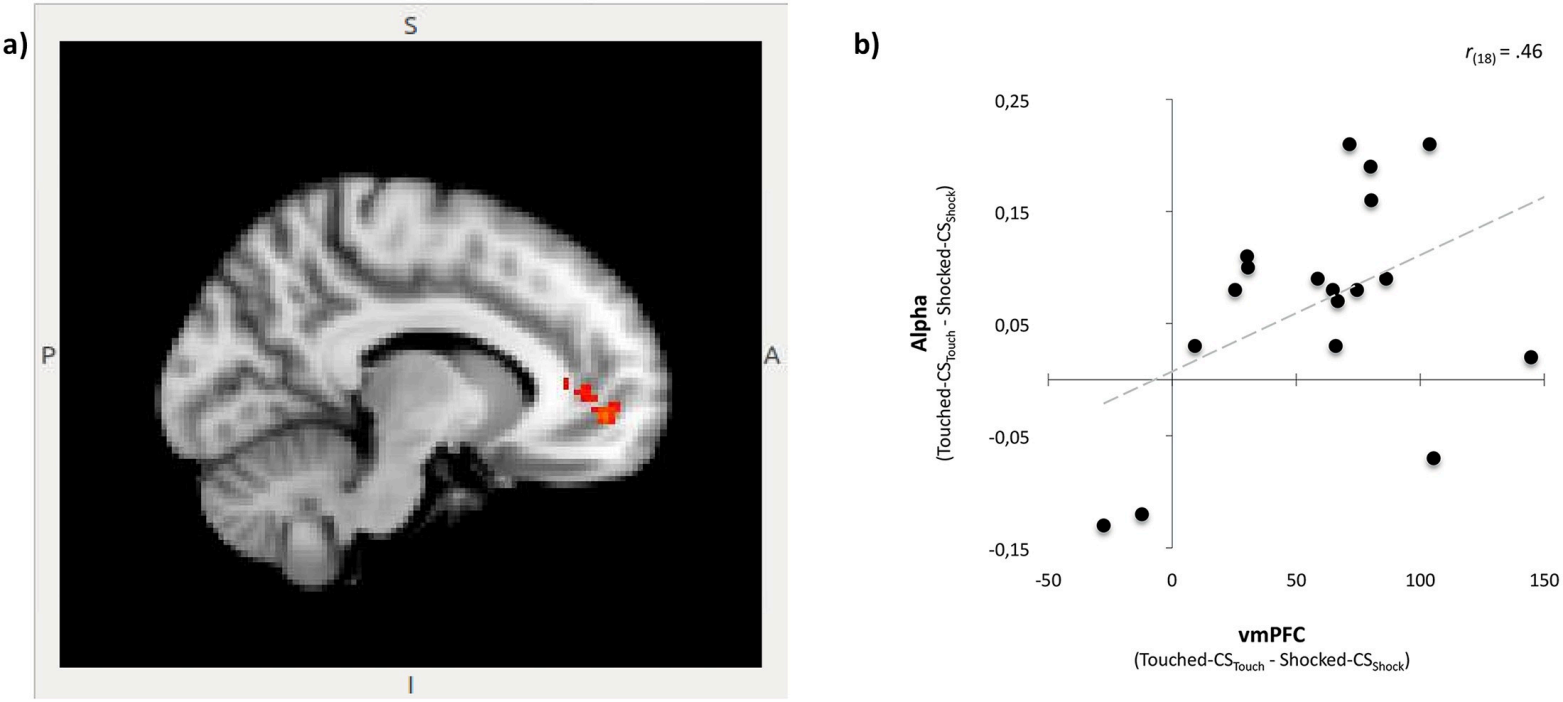
Linking the PT model valuation parameter α to brain activation. (**A**) vmPFC activation in Relief contrast. A Touched-CS_Touch_ > Shocked-CS_Shock_ contrast revealed activation within the vmPFC. We applied these masks to obtain a vmPFC ROI. (**B**) Correlation between vmPFC activity and α. Subsequent correlation analyses revealed that condition-specific activation in the vmPFC is positively related to condition-specific individual α-estimates. *Note*. CS = Conditioned Stimulus; vmPFC = ventro medial PreFrontal Cortex; PT = Prospect Theory.

Even though the above results did not reach our threshold for whole brain significance, we could still use these whole-brain contrast results to generate functional ROIs in order to test our main neural hypothesis of interest about the relationship between the incidental fear and relief from fear response and brain activity in valuation and risk areas. Specifically, because we found that relief from fear was influencing valuation and not risk-assessment in our model-based analysis of behavior, we focused on the effect of relief-of-fear on valuation in the brain, specifically in the vmPFC. To test our hypothesis that the effect of relief-of-fear on valuation would influence activity in the vmPFC we used the following procedure; first, for each subject, we extracted individual peak activation for each ROI for the Relief contrast **(**i.e., Touched_CSTouch > Shocked_CSShock**)** and individual α-estimates for Touched_CS_Touch_ as well as for Shocked_CS_Shock_ choice trials. Next, we correlated the extracted activation with the differences in α-estimates (i.e., α-Touched_CS_Touch_ minus α-Shocked_CS_Shock_). For a number of subjects, the STATA software failed to generate stable condition-specific estimates for α. Therefore, we have fewer subjects for the correlation analyses regarding the relief contrast. Results revealed that, as hypothesized, activation in the vmPFC [x = -10, y = 50, z = -4] correlated with α (*r*_(18)_ = .46, *p* = .029 (one-tailed)); see Fig 7A and 7B. These results provide evidence for a neural substrate for our main behavioral finding [43]. The remaining ROIs from the relief contrast could not be linked to α (with *r*_(18)_ ranging from.01 to.31, *p* = n.s.).

## Discussion

The precise inluence of incidental emotions on financial risk taking—emotions that are unrelated to the decision task—is still largely unclear and is not accounted for by the main formal theoretical economic models. This study examined the behavioral as well as neural impact of incidental fear on financial risk taking, using a model-based approach.

Our study renders three main findings. First, there is a clear differential behavioral effect of the CS cues—that have objectively no signalling value for the decision task at hand—after a neutral (Touch) reminder of the learned CS-US association. Specifically, decisions are faster and risk taking (economically) substantially increases when, after the experience of an US_Touch_ in the previous trial, a CS_Touch_ is observed (instead of a CS_Shock_). This suggests a relief-of-fear effect. Second, from among the main formal theoretical economic models (EUT, PT, MV), an adapted PT model allowing for a condition-dependent valuation parameter performs best in capturing this behavioral effect. When relieved of fear, the value of this parameter increases, implying an increase in the subjective value of the choice option rewards, which also predicts more risk taking with higher expected value in our environment. Third, this valuation effect is also manifested by means of an increased activity across participants in the vmPFC, a region that has previously been implicated in valuation during economic decision-making [44].

Together, these results provide some support our initial hypothesis that greater risk taking occurs when relieved of fear. A caveat is in order, though, because of the statistical weakness of the general risk-seeking test outcome (across all CS x Reminder conditions). Furthermore, we did not expect this behavioral effect to require a reminder experience of the US to make the CSs salient. Even though participants showed sustained differential pupil responding to the presentation of the CSs (with larger pupil dilation responses for CS_Shock_- than CS_Touch_-trials) indicative of successful fear conditioning, the fact that the pupil responses diminished across the trials in the decision making part of the experiment (Fig 3) may be related to this.

Because the relief-of-fear effect is best captured by the valuation parameter of the PT model, this finding points to an interactive rather than an additive effect of fear on choice. This is important because in the case of additivity, the subjective value of the experienced relief would be simply summed up together with the subjective value of the expected reward (choice outcome), which would equally affect the utility of the risky option and of the safe option, leaving choice behavior unaffected. The observed impact on the valuation of the options through a change in the valuation parameter, however, indicates an interaction between the relief of (importantly, incidental) fear and the option reward in their integration during the valuation process (see also [15]). This affects the utilities of the options differently, which makes this relief-of-fear effect behaviorally consequential, inducing more risk seeking in our environment.

Furthermore, since the change in activation of the vmPFC is measured at the time that only the CS is presented (that is, before the presentation of the lottery), while the estimated change in the valuation parameter is based on the subsequent choice behavior, the observed differential vmPFC activity is suggestive of a direct effect of fear on the valuation of the choice options. This interpretation is supported by many previous studies in which the vmPFC has been argued to play a role at the core of a neural system for valuation [45] encoding a domain-general value signal which could also include the value of emotional stimuli (creating a ‘common neural currency’) [19,20,46,44]. The finding of several human imaging studies that the mPFC gets recruited in response to a learned safety signal [40] also supports this interpretation.

Interestingly, the observed boost in risk taking when relieved of fear in this study, suggestive of exuberance, led to a higher expected payoff (Fig 5). Thus, taking expected payoff as criterion, one may rather speak of rational instead of irrational exuberance [1] in this case.

The better performance of the PT model in explaining the data, compared to the MV model and the rejected EUT model, further supports Prospect Theory as a useful tool for modeling choice behavior under uncertainty (see e.g., [30]). This study shows that this may also hold for the incorporation of the influence of incidental emotions.

No clear evidence was obtained for our complementary initial hypothesis that less risk taking would occur under incidental fear. Intriguingly, the PT-model estimation revealed some tendency for participants to become more ‘rational’ after Shocked-CS_Shock_ as compared to Shocked-CS_Touch_ by exhibiting somewhat less distorted probability weighting (Methods). Such an effect would actually help explain the observed absence of a behavioral effect. It can be shown that a less distorted probability weighting function would predict a decrease in risk seeking for gambles with a gain probability smaller than ½ but an increase for a probability larger than ½. However, although suggestive, these effects were not strong enough to reach statistical significance (see S1 Table). In future work, it would be important to determine whether such an effect would potentially manifest more robustly for gambles involving losses. Consistent with this possibility, one relevant prior did in fact report risk avoidant effects of the threat of an electric shock (compared to safe trials with no shock) for mixed gambles or gambles with only losses, but not for gambles with only gains [47]. Another factor that seems worth further exploring concerns a potential spillover effect of arousal due to having experienced strong electrical stimulation at the end of the previous trial. If this effect would hold it could potentially transform fear into anger for some participants because of stress. In that event, an effect of anger could produce an action tendency for more risk taking [12,48], and this could potentially counter the fear action tendency for less risk taking, which might help explain the absence of a simple behavioral effect [49]. It is worth noting that we observed activation in the parietal operculum in the fear contrast (Table 1). This effect could be partly related to the still experienced pain and partly to the imagined (anticipatory) pain induced by the CS_Shock_. Previous research has located the human secondary somatosensory (SII) cortex—a key region of the pain matrix—on the parietal operculum (see e.g., [50]). Furthermore, the somatosensory cortex has been suggested as a key region in the activation of somatic markers (jointly with the vmPFC, see [51]), which might help explain the (weakly significant) correlation with the probability weighting parameter (γ, see [43]). We leave these issues for future research.

This also holds for the low power for some of the statistical tests due to a small sample size that resulted from the monetary constraints from this being an fMRI study. Replication is needed to investigate the robustness of the outcomes of these tests.

In conclusion, our results show that incidental fear, and particularly the relief of such fear, can have a substantial impact on risk taking. Relief of fear appears to boost risk taking (suggestive of Greenspan’s exuberance). This behavioral effect is formally best captured by an adapted Prospect Theory model. The adaptation concerns a condition-dependent valuation of choice option rewards, which involves an increase in valuation when relieved of fear. Neurally, this is supported by related changes in the activity of the ventral medial prefrontal cortex (vmPFC).

## Supporting information

S1 Table Maximum likelihood estimates (logit) of various models based on Prospect Theory. (PNG)

S2 Table Maximum likelihood estimates (logit) of various Mean-Variance models. (PNG)

S3 Table Complete set of risky prospects used in decision-making task. (PDF)
