# Supplementary figures and images for: Relief from incidental fear evokes exuberant risk taking
Source: PLoS One. 2019 Jan 24;14(1):e0211018. doi: 10.1371/journal.pone.0211018 (PMC6345498; doi:10.1371/journal.pone.0211018)

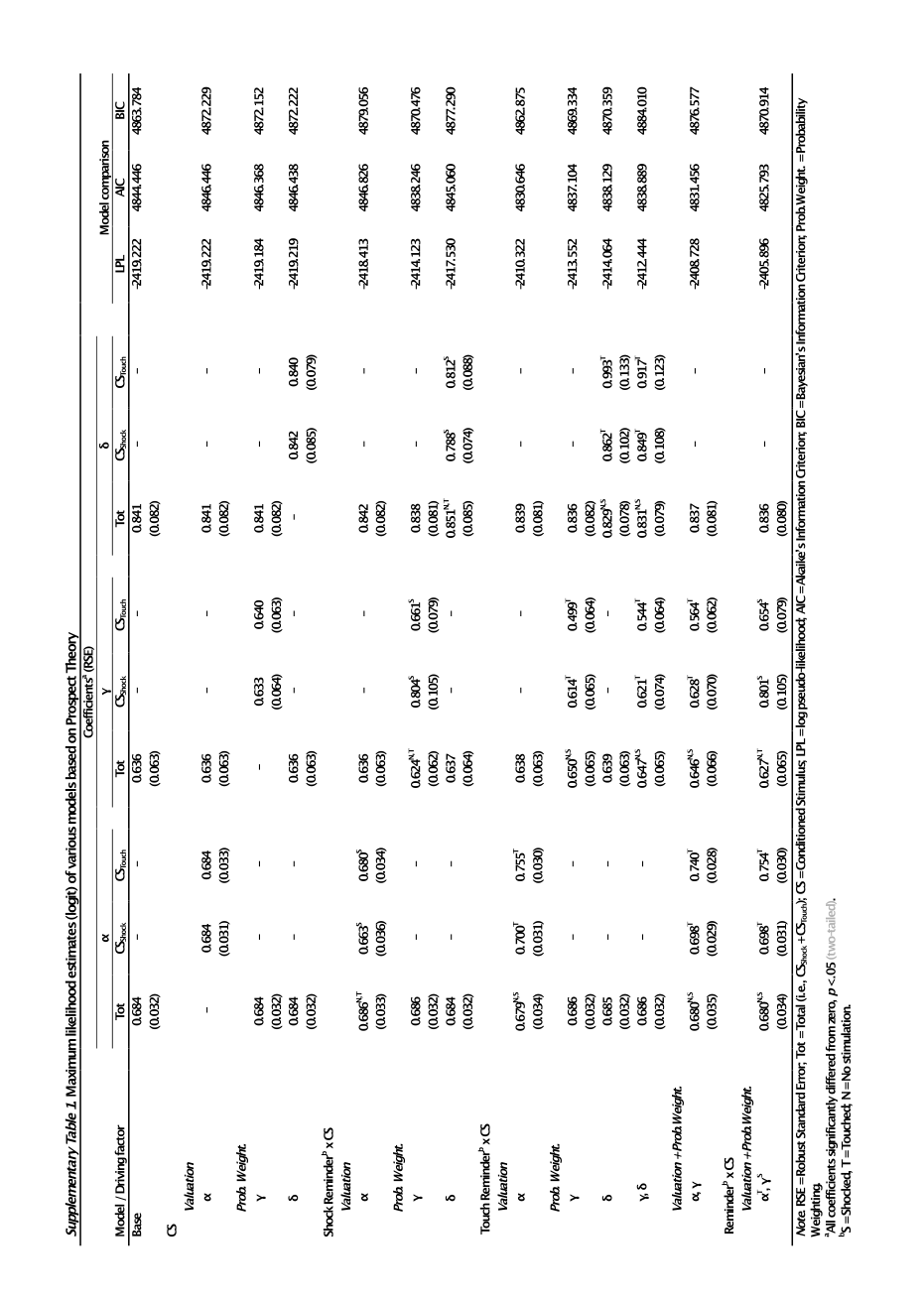

Supplement: S1 Table — (PNG) [file pone.0211018.s001.png]

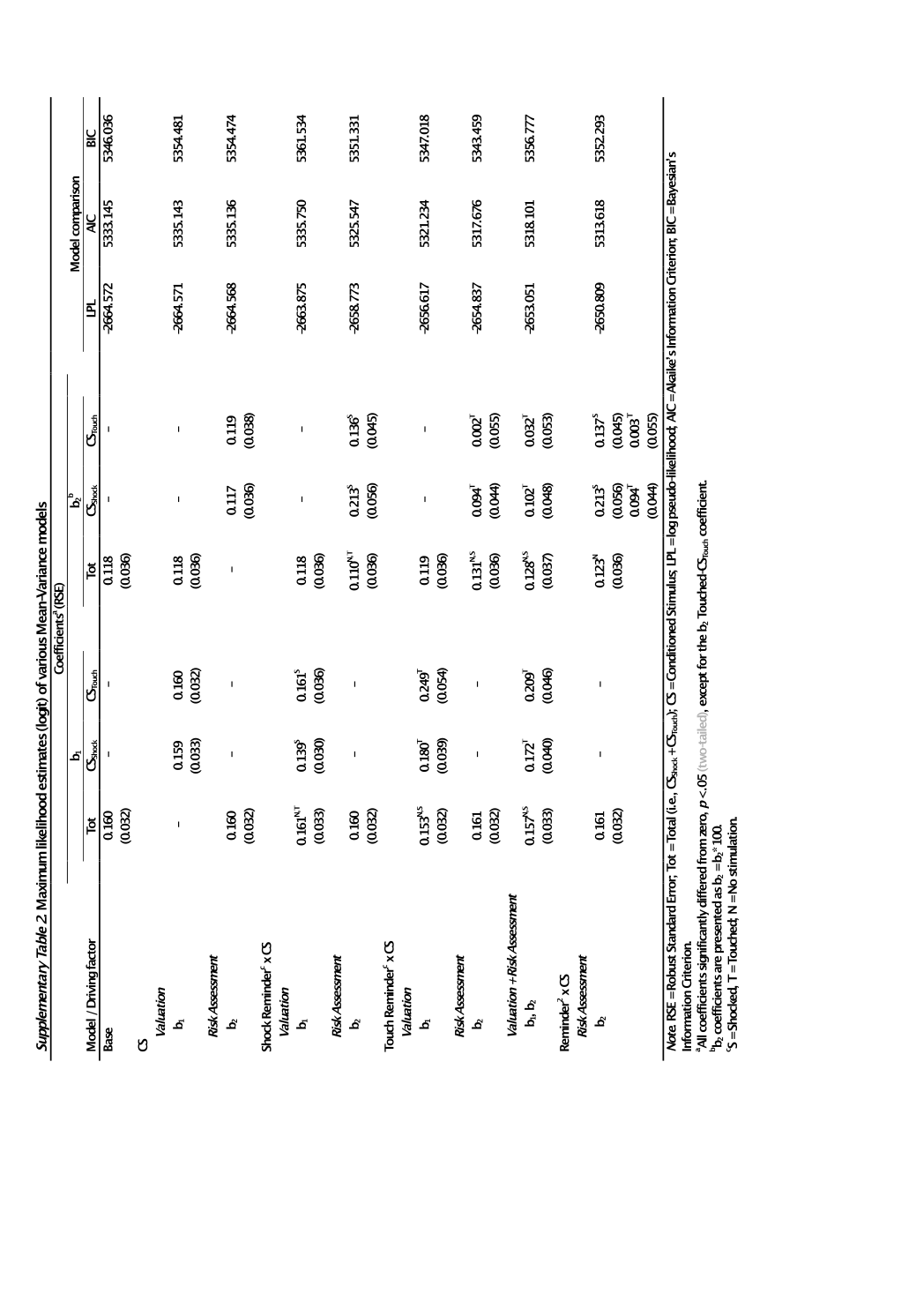

Supplement: S2 Table — (PNG) [file pone.0211018.s002.png]
