# Supplementary material for: Relief from incidental fear evokes exuberant risk taking
Source: PLoS One. 2019 Jan 24;14(1):e0211018. doi: 10.1371/journal.pone.0211018 (PMC6345498; doi:10.1371/journal.pone.0211018)
Supplement: S3 Table — (PDF) [file pone.0211018.s003.pdf]

Supplementary Table 3. Complete set of risky prospects used in decision-making task

Risky prospect	Probability (%)	Magnitude (€)	Probability (%)	Magnitude (€)
1	91	11	9	0
2	85	16	15	0
3	80	18	20	0
4	50	20	50	0
5	75	20	25	0
6	25	23	75	0
7	85	25	15	0
8	75	27	25	0
9	12,5	30	87,5	0
10	40	30	60	0
11	50	30	50	0
12	60	30	40	0
13	90	30	10	0
14	25	33	75	0
15	45	33	55	0
16	75	33	25	0
17	50	35	50	0
18	50	36	50	0
19	55	36	45	0
20	25	40	75	0
21	5	45	95	0
22	95	45	5	0
23	50	46	50	0
24	25	60	75	0
25	20	70	80	0
26	12,5	80	87,5	0
27	25	80	75	0
28	15	93	85	0

Note. Each risky prospect had to be considered against a safe prospect, which entailed a guaranteed gain of €10.
